# Does extracorporeal shockwave lithotripsy therapy affect thiol-disulfide homeostasis?

**DOI:** 10.12669/pjms.345.15823

**Published:** 2018

**Authors:** Aliseydi Bozkurt, Cuma Mertoglu, Mehmet Karabakan, Gulsah Siranli, Emine Feyza Yurt, Ozcan Erel

**Affiliations:** 1 Aliseydi Bozkurt, Department of Urology, Faculty of Medicine, Erzincan University, Erzincan, Turkey; 2 Cuma Mertoglu, Department of Clinical Biochemistry, Faculty of Medicine, Erzincan University, Erzincan, Turkey; 3 Mehmet Karabakan, Department of Urology, Erzincan University, Erzincan, Turkey; 4 Gulsah Siranli, Department of Clinical Biochemistry, Faculty of Medicine, Erzincan University, Erzincan, Turkey; 5 Emine Feyza Yurt, Department of Clinical Biochemistry, Faculty of Medicine, Yildirim Beyazit University, Ankara, Turkey; 6 Ozcan Erel, Department of Clinical Biochemistry, Faculty of Medicine, Yildirim Beyazit University, Ankara, Turkey

**Keywords:** Extracorporeal shockwave lithotripsy, Thiol/disulfide, Kidney stone, Oxidative stress, Ischaemia modified albumin

## Abstract

**Objective:**

Extracorporeal Shockwave Lithotripsy (ESWL) is a non-invasive method that is effective at crushing stones in the upper urinary tract. Disturbance of the thiol/disulfide homeostasis, in favor of the disulfide, has been shown to be involved in the disease pathogenesis.

**Methods:**

A total of 36 individuals that underwent ESWL had blood samples collected before ESWL (0hrs), 6hrs, and one week after the ESWL. Sera native and total as wells as disulfide amount was measured using an automated method sodium borohydrate (NaBH_4_) reduction. In addition, Ischemia Modified Albumin (IMA) levels were measured using colorimetric assay method.

**Results:**

Native thiol level was reduced at the 6th hour following ESWL compared to baseline. While the ratios of disulfide level, Disulfide/Total Thiol (DTT), Disulfide/Native Thiol (DNT) and IMA level were increased at the 6th hour following ESWL compared to baseline, they were found to be similar with their baseline values at the end of 1st week. Total thiol and native /total thiol did not show any significant change.

**Conclusions:**

ESWL treatment disrupts thiol/disulfide homeostasis and the structure of albumin at the acute term. Therefore, it increases protein oxidation and leads to increased oxidative stress. However, this state is transient and returns to normal within the proceeding days.

## INTRODUCTION

The Clinical Guidelines of the European Association of Urology (EAU) endorse the use of Extracorporeal Shockwave Lithotripsy (ESWL) together with retrograde intra-renal surgery for treating kidney stones (<2 cm).^1^ ESWL is a non-invasive method that is effective at crushing stones in the upper urinary tract.^2^ However, shock waves cause tissue injury in exposed kidney.^3^

Thiols are organic compounds containing a sulfhydryl group (–SH).^4^ When oxidized, thiols (-SH) form disulphide (-S-S-) bonds or bridges that can be intra- or intermolecular scaffolds. This phenomenon is especially true under Oxidative Stress (OS) where the cysteine residues undergo complete oxidation and form these disulfide crosslinks. In addition, these oxidized crosslinks are reversible and can be reduced back to thiols or mercaptans, which completes the thiol–disulphide homeostasis.^5^ Disturbance of thiol/disulfide homeostasis in favor of disulfide has been shown to impact the disease pathogenesis.^6^ In various conditions such as ischemia, hypoxia, metabolic acidosis, and augmented OS, the structure of albumin alters its form to Ischemia-Modified Albumin (IMA). To date, IMA is the only clinical test permitted by the Food and Drug Administration (FDA) for ischemia.^7^,^8^

In some recent studies, it has been reported that ESWL treatment increases OS, and that this can be prevented by antioxidant treatments.^9^-^12^ Measurements of thiol and disulfide levels have become available with a reliable, convenient, and inexpensive assay method that has recently been developed.^6^ In this study, we investigated the effect of ESWL treatment on protein oxidation, specifically OS, by measuring serum thiol/disulfide levels with this method.

## METHODS

A total of 36 individuals (13 female and 23 male) that underwent ESWL in our hospital for the treatment of kidney stones were included in this prospective, controlled, and single-centered study. The lithotripter used was an EMD E-1000 electromagnetic ESWL. Full approval was received by the local ethical committee (number: 804.01-E.32937, date: 26/07/2017). In addition, all the participants were screened and informed consent was obtained.

### Inclusion criteria

- Urolithiasis diagnosed by ultrasound examination and computed tomography.
- ESWL procedure performed for the first time or repeated (with at least 6-month interval between subsequent procedures).
- Maximum crushed stone size of 20 mm.
- Normal renal function confirmed by serum creatinine levels and estimated glomerular filtration rate values.

The patients that had an acute infection, a solitary kidney, congenital renal anomalies, urinary tract disorders such as pyelonephritis, diabetes, hypertension, cardiovascular disease, or any other known chronic diseases as well as those that had previously undergone renal surgery were excluded from the study.

### Biochemical Analyses

The serum samples were obtained from patients before ESWL (0hrs), at six hours, and seven days after the ESWL and immediately centrifuged, aliquoted, and stored indefinitely at -80 °C until the analyses day. The total concentrations of serum creatinine, urea, glucose and albumin were analyzed using a Beckman Coulter Olympus AU 2700 system.

Native/total thiol as well as the ratio of disulfide/native or total thiol were analyzed using an automated method as previously described.^6^ Briefly, the oxidized disulfides (–S–S–) were reduced to mercaptans (–SH) using NaBH_4_. Following this procedure, the residual NaBH_4_ materials were entirely removed from the biochemical environment with formaldehyde. Ellman’s reagent was performed to calculate the amount of total thiol. Total disulfide content was calculated the difference between the half the amount of total and native thiols. In this way, the native and total thiol as well as disulfides were calculated, and the percentage of disulfide/total thiol, disulfide/native thiol and native-to-total thiol was collected. Serum IMA levels were calculated using a colorimetric assay method described previously.^7^ In brief, 200 µL of a subject serum was added to 50 µL of 0.1% CoCl2 followed by mixing and 10 minutes of incubation in the dark at 37ºC. Then, a total of 50 µL dithiothreitol was added as a coloring agent. After two minutes of incubation, 1mL of 0.9% NaCl was added and the blank control with reducing agent was similarly prepared. Absorbance at 470 nm was used to obtain readings on the spectrophotometer. IMA was recorded as absorbance units (ABSUs). Each sample was measured in duplicate and the mean value was reported.

### Statistical Analysis

Data were analyzed using SSPS Software for Windows v 18.0 (SPSS Inc., Chicago, IL, USA) and the mean, standard deviation, median, and 95 % CI number were generated in the three groups. The Kolmogorov-Smirnov test was used to check normality. The normal distribution analysis was performed using one-way ANOVA and paired a posthoc test. If the samples were non-normally distributed, a Kruskal-Wallis and Mann-Whitney-U test was used. A p-value of less than 0.05 was considered statistically significant.

## RESULTS

**Fig.1:**
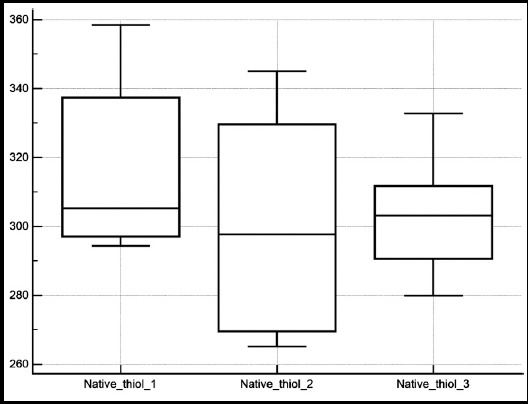
The change in mean native thiol levels measured before ESWL (native thiol 1) after 6 hour (native thiol 2), and 7th day (native thiol 3) from ESWL.

**Fig.2:**
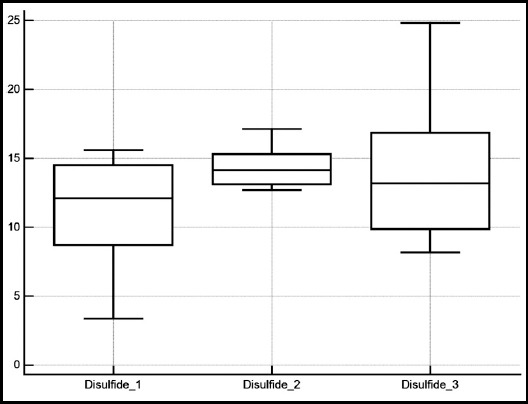
The change in mean disulfide levels measured before ESWL (disulfide 1), after 6 hour (disulfide 2), and 7th day (disulfide 3) from ESWL.

**Fig.3:**
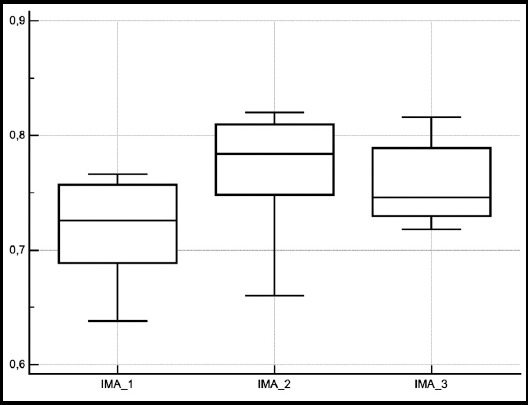
The change in median ischemia-modified albumin levels measured before ESWL (IMA 1), after 6 hour (IMA 2), and 7th day (IMA 3) from ESWL.

The patient demographics and data regarding the treatment procedure are shown in Table-I and II. Urea, creatinine and albumin levels were found to be similar when post-ESWL values compared to baseline (Table-III). Native thiol level was reduced at the 6th hour following ESWL compared to baseline. However, native thiol levels measured at the end of the 7th day was similar with baseline (Fig.1). While disulfide level, disulfide/total thiol, disulfide/native thiol, and IMA level were increased at the 6th hour following ESWL compared to baseline, they were found to be similar with their baseline values at the end of 7th day (Fig.2). Total and native thiol to total thiol was not statistically significant. IMA level was increased at the 6th hour following ESWL compared to baseline, whereas IMA level at 1st week was similar with both baseline and 6th hour levels (Fig.3).

**Table-I:** Demographic data of the patients enrolled into the study.

Parameter	Mean±SD
Age	41±15
Male	23 (63.8 %)
Female	13 (36.2 %)
BMI (kg/m^2^)	26.2±4.8
Lenght	164.1±6.35
Weight	71.6±13.2

Data are presented as mean±SD for continuous variables and as number (percentage) for categorical variables. ***Abbreviations:*** BMI: Body mass index.

**Table-II:** ESWL data of the patients enrolled into the study.

Parameter	Mean±SD
Previous stone treatment (> 6 month ESWL)	7 (19.4 %)
Parenchymal thickness (mm)	16.1±4.2
Mean hounsfield units	885±43.6
Pulses generated	2648 ±245
Wawes	77.2±1.69
The mean value of energy (joule)	16.17 ±3.19
Grade of hydronephrosis None	6 (16.6 %)
Mild	21 (58.3 %)
Moderate	7 (19.5 %)
Severe	2 (5.6 %)
*Stent post ESWL*	
No	
Stone location Pelvis	21(58.4 %)
Middle Calix	10(27.8%)
Upper calix	3(8.3 %)
Lower Calix	2(5.5%)
Stone Size (mm^2^)	10.5.2±3.4

Data are presented as mean± SD for continuous variables and as number (percentage) for categorical variables. ***Abbreviations:*** ESWL: Extracorporeal shockwave lithotripsy

**Table-III:** Labaratory findings of the study population.

Parameter	Before ESWL 0. hour Mean 95% CI	After ESWL 6. hour Mean 95% CI	After ESWL 7. day Mean 95% CI	P value	Group, p value
Native thiol (µmol/L)	325±39.7	316±49.1	302±19.3	0.003	1-2, 0.021^a^
					1-3, 0.377
	311-339	299-333	278-327		2-3, 0.881
Disulfide (µmol/L)	12.3±5.29	15.2±5.85	14.1±6.40	0.001	1-2, 0.009^b^
					2-3, 0.940
	10.3-14.2	13.3-17.5	6.20-22.1		1-3, 0.442
Total thiol (µmol/L)	351±41.7	346±52.5	331±19.5	0.175	1-2, 0.225
					1-3, 0.467
	336-365	328-364	306-355		2-3, 0.870
Native thiol/ Total thiol (%)	92.7±3.2	91.3±3.36	91.4±3.5	0.012	1-2, 0.070
					1-3, 0.407
	91.6-93.8	90.2-92.5	87.1-95.8		2-3, 0.865
Disulfide/ Total thiol (%)	3.61±1.61	4.46±1.60	3.79±1.94	0.001	1-2, 0.004^b^
					2-3, 0.865
	3.05-4.18	3.88-5.04	1.75-5.83		1-3, 0.408
Disulfide/ Native thiol (%)	3.96±1.94	4.97±1.96	4.72±2.15	0.001	1-2, 0.005^b^
					1-3, 0.398
	3.28-4.64	4.26-5.67	2.04-7.40		2-3, 0.907
Albumin (g/dL)	4.58±0.12	4.58±0.14	4.64±0.06	0.126	1-2, 0.855
					1-3, 0.375
	4.54-4.62	4.53-4.63	4.55-4.72		2-3, 0.578
IMA (ABSUs)	0.738*	0.779*	0.746*	0.001	1-2, 0.007^c^
					1-3, 0.187
	0.698-0.779	0.739-0.790	0.710-0.807		2-3, 0.996
Urea (mg/dL)	30.6±10.6	32.1±15.2	31.5±12.5	0.341	1-2, 0.258
					1-3, 0.455
	26.9-34.4	23.1-38.2	24.6-36.2		2-3, 0.312
Creatinine (mg/dL)	0.97±0.17	1.05±0.21	1.01±0.20	0.428	1-2, 0.355
					1-3, 0.456
	0.91-1.03	0.89-1.15	0.92-1.10		2-3, 0.475
GFR (mL/min)	90.7±18.0	86.8±20	88.20	0.544	1-2, 0.487
					1-3, 0.658
	84.3-97.1	82.1-100.1	84.1-98.5		2-3, 0.489

* Median value

a p < 0.05 by paired samples t-test,

b p < 0.01 by independent samples t-test.

c p < 0.01 by Mann Withney - U test.

## DISCUSSION

ESWL is the method of choice for the treatment of stone in the urinary tract. Despite its proven safety and efficacy, multiple reports have shown that complications can arise from ESWL.^3^,^13^

In their study with patients who have underwent ESWL treatment, Aksoy et al.^10^ found increased levels of the antioxidant enzymes at the 1st hour following ESWL compared to baseline. Whereas on the 5th day, the levels of the same enzymes were found to be similar with baseline values. Interestingly, the level of malondialdehyde (an indicator of OS) was increased in the 1st hour following ESWL, but was similar on the 5th day when compared to baseline. Another clinical study 14 showed that ESWL procedure led to increased levels of nitrate, which is another OS indicator. In their study, Sanyuce et al.^9^ reported increased malondialdehyde levels and histopathological changes in kidneys following experimental ESWL.

Also, urinary levels of renal injury markers such as the cortical tubular enzyme and proteins including NAG, retinol-binding protein, and *β_2_* - microglobulin were shown to be increased early after ESWL and reduced to normal within the proceeding days.^11^,^15^ In another study, levels of homocysteine, which is an indicator of oxidative stress, were shown to be increased following ESWL, and remain elevated even after three months when compared to baseline.^16^

Previous studies have also shown that antioxidant treatments can reduce OS caused by ESWL, and have favorable effects on kidney functions.^11^,^12^ Similar to all these reports, the present study has shown that ESWL treatment causes a transient augmentation of OS. Thiol/disulfide homeostasis is disturbed early after ESWL treatment. However, this state is transient and returns to normal in time. Indeed, level of native thiol, which has antioxidant property, was reduced at the 6th hour following ESWL. However, it increased again close to its baseline values at the end of 7th day. Also, disulfide, disulfide/total thiol, disulfide/native thiol and IMA levels showed an increase at the 6th hour following ESWL. However, they reduced close to their baseline values at the 7th day. All these results indicate that ESWL causes a transient increase in OS, which resolves in time.

Disturbance of thiol/disulfide homeostasis in favor of the disulfide as well as increased IMA formation are among the important factors that are responsible for the pathophysiology of systemic diseases including diabetes^17^, hypertension^18^ kidney and prostate disorders including chronic kidney disease^19^ and prostate cancer.^20^

Wozdiak et al.^21^ studied 12 patients who underwent ESWL treatment, and they found similar levels of carbonyl groups and protein thiol groups in measurements before and one day after ESWL. This finding, which is not consistent with present report, may be an effect of the small patient size. Moreover, the fact that disulfide and total thiol levels were also analyzed in addition to the thiol groups adds more value to the present study.

In the present study, the reason for the disturbance in the thiol/disulfide homeostasis and increased OS may be due to the renal vascular hemorrhage, inflammation, and ischemia caused by ESWL treatment, which is consistent with previous reports.^9^,^22^ IMA level was increased early after ESWL, which was considered to be consistent with ischemia. Indeed, Doppler studies have demonstrated the presence of an ischemic state early after ESWL, which was found to resolve within the proceeding days.^23^ Furthermore, high amounts of blood are filtered through the kidneys. Therefore, ESWL may also affect blood cells.^10^

### Limitations of the Study

The relatively small patient cohort is a limitation to the current report. Therefore, future studies should be aimed to sample larger population and to investigate the molecular underpinnings of OS and ischemic state caused by ESWL treatment.

## CONCLUSIONS

On the whole current report demonstrates that ESWL treatment causes a disturbance in the thiol/disulfide homeostasis and in the structure of albumin in the acute term, which increases protein oxidation and OS. However, this state is transient, and returns to normal within the proceeding days.

### Authors’ Contribution

**AB:** Conceiving, Study designing, Data collection, Manuscript writing, Review and final analysis.

**CM, MK, GS, EFY, OE:** Data collection, Data interpretation, Statistical analysis, Discussion writing and final analysis.
